# TRAV1-2^+^ CD8^+^ T-cells including oligoconal expansions of MAIT cells are enriched in the airways in human tuberculosis

**DOI:** 10.1038/s42003-019-0442-2

**Published:** 2019-06-05

**Authors:** Emily B. Wong, Marielle C. Gold, Erin W. Meermeier, Bongiwe Z. Xulu, Sharon Khuzwayo, Zuri A. Sullivan, Eisa Mahyari, Zoe Rogers, Hénrik Kløverpris, Prabhat K. Sharma, Aneta H. Worley, Umesh Lalloo, Prinita Baijnath, Anish Ambaram, Leon Naidoo, Moosa Suleman, Rajhmun Madansein, James E. McLaren, Kristin Ladell, Kelly L. Miners, David A. Price, Samuel M. Behar, Morten Nielsen, Victoria O. Kasprowicz, Alasdair Leslie, William R. Bishai, Thumbi Ndung’u, David M. Lewinsohn

**Affiliations:** 1 Africa Health Research Institute, KwaZulu-Natal, South Africa; 2 Division of Infectious Diseases, Massachusetts General Hospital, Boston, MA USA; 3 Harvard Medical School, Boston, MA USA; 4 Division of Infection and Immunity, University College London, London, UK; 5 Department of Pulmonary & Critical Care Medicine, Oregon Health & Science University, Portland, OR USA; 6 VA Portland Health Care System, Portland, OR USA; 7 Department of Molecular Microbiology & Immunology, Oregon Health & Science University, Portland, OR USA; 8 Division of Bioinformatics and Computational Biology (BCB), Department of Medical Informatics and Clinical Epidemiology (DMICE), Oregon Health & Science University, Portland, OR USA; 9 Institute for Immunology and Microbiology, University of Copenhagen, Copenhagen, Denmark; 10 Durban University of Technology, Durban, South Africa; 11 Department of Pulmonology, Inkosi Albert Luthuli Hospital, Durban, South Africa; 12 Department of Pulmonology & Critical Care, Nelson R. Mandela School of Medicine, University of KwaZulu-Natal, Durban, South Africa; 13 Department of Cardiothoracic Surgery, Nelson R. Mandela School of Medicine, University of KwaZulu-Natal, Durban, South Africa; 14 Centre for AIDS Programme of Research in South Africa (CAPRISA), Durban, South Africa; 15 Institute of Infection & Immunity, Cardiff University School of Medicine, Cardiff, Wales UK; 16 Human Immunology Section, Vaccine Research Center, National Institute of Allergy and Infectious Diseases, National Institutes of Health, Bethesda, Maryland USA; 17 Department of Microbiology and Physiological Systems, University of Massachusetts Medical School, Worcester, MA USA; 18 Center for Biological Sequence Analysis, Department of Bio and Health Informatics, Technical University of Denmark, Lyngby, Denmark; 19 Instituto de Investigaciones Biotecnológicas, Universidad Nacional de San Martín, Buenos Aires, Argentina; 20 HIV Pathogenesis Programme, Doris Duke Medical Research Institute, Nelson R. Mandela School of Medicine, University of KwaZulu-Natal, Durban, South Africa; 21 The Ragon Institute of MGH, MIT, and Harvard, Harvard Medical School, Cambridge, MA USA; 22 Division of Infectious Diseases, Johns Hopkins University School of Medicine, Baltimore, MD USA; 23 Max Planck Institute for Infection Biology, Berlin, Germany

**Keywords:** Mucosal immunology, T-cell receptor, Tuberculosis

## Abstract

Mucosal-associated invariant T (MAIT) cells typically express a TRAV1-2^+^ semi-invariant TCRα that enables recognition of bacterial, mycobacterial, and fungal riboflavin metabolites presented by MR1. MAIT cells are associated with immune control of bacterial and mycobacterial infections in murine models. Here, we report that a population of pro-inflammatory TRAV1-2^+^ CD8^+^ T cells are present in the airways and lungs of healthy individuals and are enriched in bronchoalveolar fluid of patients with active pulmonary tuberculosis (TB). High-throughput T cell receptor analysis reveals oligoclonal expansions of canonical and donor-unique TRAV1-2^+^ MAIT-consistent TCRα sequences within this population. Some of these cells demonstrate MR1-restricted mycobacterial reactivity and phenotypes suggestive of MAIT cell identity. These findings demonstrate enrichment of TRAV1-2^+^ CD8^+^ T cells with MAIT or MAIT-like features in the airways during active TB and suggest a role for these cells in the human pulmonary immune response to *Mycobacterium tuberculosis*.

## Abstract (web summary)

Emily Wong et al. observe that Mycobacterium tuberculosis (Mtb) infection induces recruitment and expansion of a CD8^+^ TRAV1-2^+^ T-cell population in the airways of human patients. T cell receptor analysis showed expansion of this cell population, including oligoclonal MAIT cells, in sites where Mtb antigens are present, suggesting they act as sentinels of pulmonary infection.

## Introduction

Mucosal-associated invariant T (MAIT) cells are unconventional lymphocytes that use semi-invariant T cell receptor-alpha (TCRα) chains to recognize non-peptide small molecule ligands presented by the HLA-Ib molecule MR1^1–6^. In mice, MAIT cells have been shown to play a protective role in models of respiratory infection^7–10^. In humans, MAIT cells are abundant in the peripheral blood of healthy individuals, where they produce cytolytic enzymes and pro-inflammatory cytokines and typically express a TRAV1-2^+^ TCRα chain and the CD8 coreceptor^1–4,11–13^. MAIT cells are depleted in the blood of humans with TB^4,5,14^. However, little is known about the function and phenotype of MAIT cells in the human lung, especially in the setting of pulmonary tuberculosis (TB). We postulated that MAIT cells are recruited to and/or expand at sites where *Mycobacterium tuberculosis* (Mtb) antigens are present, potentially acting as sentinels of infection in the respiratory mucosa.

Here we report that a population of pro-inflammatory TRAV1-2^+^ CD8^+^ T cells are present in the airways and lungs of healthy individuals and are enriched in bronchoalveolar fluid of patients with active pulmonary TB. Some of these cells demonstrate MR1-restricted mycobacterial reactivity, phenotypic features and/or TCRα chain usage suggestive of MAIT cell identity. We conclude that TRAV1-2^+^ CD8^+^ T cells with MAIT or MAIT-like features are oligoclonally expanded in the airways during active TB, suggesting that they play a role in the human pulmonary immune response to *Mycobacterium tuberculosis*.

## Results

### TRAV1-2^+^ CD8^+^ T-cells in human lung and intestine tissues

**Fig. 1:**
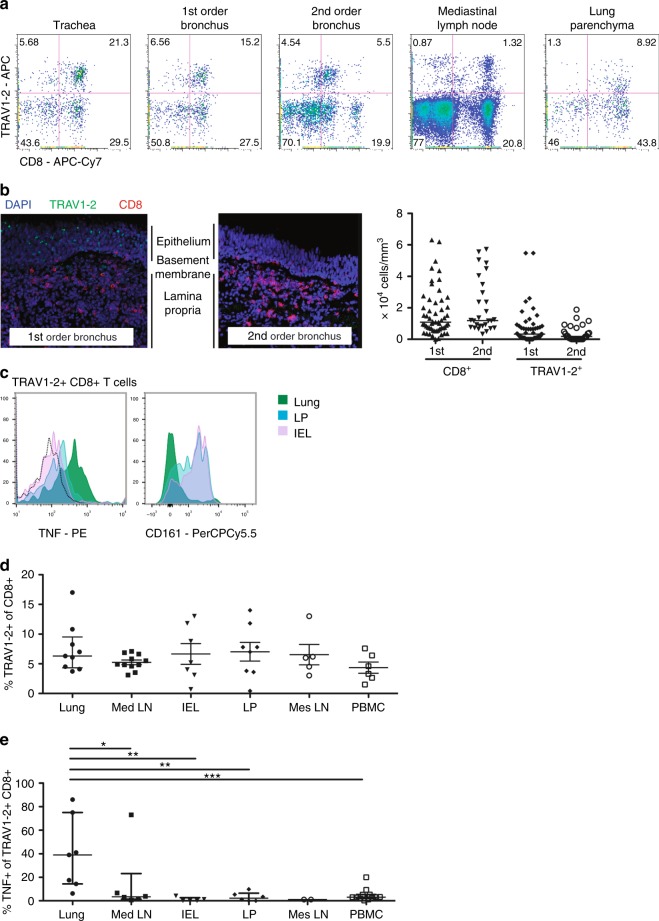
TRAV1-2^+^ CD8^+^ T cells from the lung but not the intestine of healthy organ donors respond to mycobacterial infection by producing TNF. **a** Dot plots showing the frequency of TRAV1-2^+^ CD8^+^ T cells among live CD3^+^ cells in the indicated tissue samples from one donor. **b** Tissue sections from the 1st and 2nd order bronchi were obtained from healthy individuals (*n* = 3 biologically independent samples). Immunohistochemistry was performed to quantify CD8^+^ (median 1.6 × 10^4^ vs. 2 × 10^4^ cells /mm^3^) and TRAV1-2^+^ cells (7,000 vs. 4,000 cells/mm^3^, Supplementary Data). Representative sections from 1st and 2nd order bronchi are depicted (left), showing CD8^+^ cells (red), TRAV1-2^+^ cells (green) and cell nuclei (DAPI; blue). **c** Histograms depicting TNF production (left) and CD161 expression (right) by TRAV1-2^+^ CD8^+^ T cells from matched lung parenchyma (green), small intestine lamina propria (LP; blue) and the small intestinal intraepithelial layer (IEL; violet) after overnight stimulation with *M. smegmatis-*infected antigen-presenting cells (dotted black line indicates the unstimulated control). **d** Frequency of TRAV1-2^+^ cells among CD8^+^ T cells from lung (*n* = 9 biologically independent samples), mediastinal lymph node (Med LN; *n* = 11 biologically independent samples), IEL (*n* = 7 biologically independent samples), LP (*n* = 8 biologically independent samples), mesenteric lymph node (Mes LN, *n* = 5 biologically independent samples), and peripheral blood (PBMC; *n* = 6 biologically independent samples, Supplementary Data). Medians and interquartile ranges are displayed. **e** Frequency of TNF-producing TRAV1-2^+^ CD8^+^ T cells after exposure to *M. smegmatis*-infected antigen-presenting cells: lung (*n* = 7 biologically independent samples), Med LN (*n* = 6 biologically independent samples), IEL (*n* = 5 biologically independent samples), LP (*n* = 6 biologically independent samples), Mes LN (*n* = 2 biologically independent samples), PBMC (*n* = 12 biologically independent samples, Supplementary Data). From top to bottom, *P* = 0.035, 0.0025, 0.0023 and 0.0005 (Mann–Whitney *U* test). Medians and interquartile ranges are displayed

To explore the role of MAIT cells in healthy mucosal tissues, we first determined the frequency of TRAV1-2^+^ CD8^+^ cells in the respiratory tract of an individual organ donor (Fig. 1a). Dramatic enrichment was observed in the trachea, where nearly half of all CD8^+^ T cells expressed TRAV1-2 (Fig. 1a). TRAV1-2^+^ cells were also enriched in the proximal and distal bronchi (35 and 22% of CD8^+^ T cells, respectively) and in the lung parenchyma (17% of CD8^+^ T cells), relative to the draining mediastinal lymph node where the frequency (6% of CD8^+^ T cells) approximated levels typically found in peripheral blood^11,15^. To determine the anatomical localization of TRAV1-2^+^ CD8^+^ cells in the airway, we used immunohistochemistry to quantify CD8^+^ and TRAV1-2^+^ cells in 1st and 2nd order bronchial sections from three additional organ donors (Fig. 1b, left). Although the number of CD8^+^ cells was similar in tissue sections from the proximal and distal airways, TRAV1-2^+^ cells were more frequent in the proximal compared to distal airway (Fig. 1b, right). As expression of TRAV1-2^+^ TCRs is insufficient to define MAIT cells, we also performed ex vivo functional assays in which cytokine-production by TRAV1-2^+^ CD8^+^ cells upon exposure to HLA mismatched *M. smegmatis*-infected antigen-presenting cells is used to define mycobacterial-reactive MAIT cells^4,12,16,17^. In a single donor for whom paired tissues were available, we evaluated lymphocytes from lung parenchyma, the small intestinal lamina propria (LP), and the small intestinal intraepithelial lymphocytes (IEL) for *M. smegmatis*-dependent release of the pro-inflammatory cytokine TNF. Interestingly, TNF-producing TRAV1-2^+^ cells were found only in the lung (Fig. 1c, left). It is also notable that CD161, a C-type lectin highly expressed on peripheral MAIT cells^11,13,17^, was not detected on TRAV1-2^+^ CD8^+^ T cells from the lung but was found in abundance on small intestinal TRAV1-2^+^ CD8^+^ T cells (Fig. 1c, right). We next compared the frequencies of TRAV1-2^+^ and TNF-producing cells in the lung (*n* = 9) and intestinal mucosa (*n* = 8, unmatched samples) where MAIT cells were initially found to be enriched^3^. The frequencies of TRAV1-2^+^ CD8^+^ T cells were similar across mucosal sites, associated lymphoid tissues and unmatched peripheral blood samples (*n* = 6) (Fig. 1d). In contrast, significantly higher frequencies of TRAV1-2^+^ cells from the lung produced TNF in response to *M. smegmatis*-infected cells compared with TRAV1-2^+^ cells from lymphoid tissues, small intestine, or peripheral blood (*P* = 0.035, 0.0025, 0.0023 and 0.0005 (Mann–Whitney *U* test), Fig. 1e). Cell yields from these tissues were insufficient to establish functional dependence on MR1 as has been shown previously with this assay^4^. Nonetheless, these data demonstrate that mycobacterial stimulation results in TNF production by donor-unrestricted, lung resident TRAV1-2^+^ CD8^+^ T cells.

### TRAV1-2^+^ CDR3α usage in Mtb-infected lung tissue

**Fig. 2:**
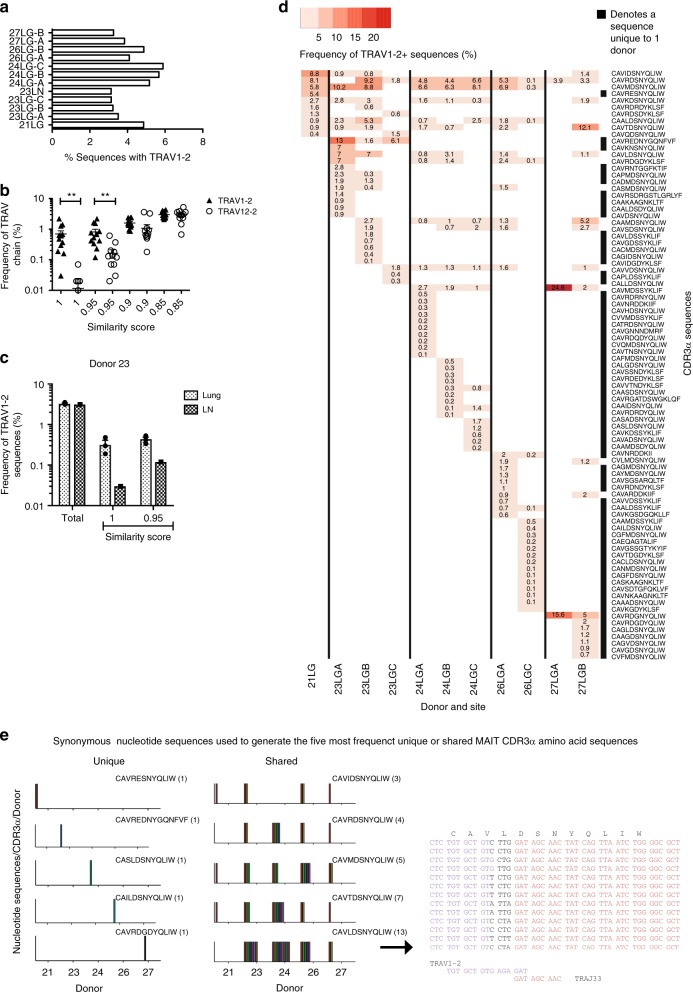
Expansions of MAIT cell-consistent CDR3α‘s are present in tuberculous lung granulomas. **a** Frequency of TRAV1-2^+^ sequences as a percentage of all productive TCRα sequences. In some cases, multiple areas of tissue were sampled, ranging from closest (A) to furthest (C) from the site of disease. **b** MAIT cell TCRα sequences are consistent with similarity scores of 0.95 and 1. Each symbol represents the frequency of TRAV1-2^+^ or TRAV12-2^+^ sequences within each similarity score for each donor sample (*n* = 12 biologically independent samples). **c** Frequency of total TRAV1-2^+^ sequences or those with similarity scores of 0.95 and 1 in the lung (*n* = 3 biologically independent samples) and mediastinal lymph node (LN, 1 sample) from donor 23. Height represents mean, error bars represent standard error. **d** Frequencies among TRAV1-2^+^ sequences of the top 10 public and private MAIT cell CDR3α sequences (MAIT Match score ≥0.95) across individual donors and lung samples. **e** Variation in the number of synonymous nucleotide sequences encoding the five most frequent private (left) and public (right) MAIT cell CDR3α amino acid (aa) sequences from all samples displayed in Fig. 3d. For each aa sequence, each colored bar represents a different nucleotide sequence. The 13 different nucleotide sequences used to generate the shared MAIT cell CDR3α aa sequence CAVLDSNYQLIW are displayed. Text color represents nucleotide origin: purple (TRAV), black (TRAD or n insertion), red (TRAJ). LG lung granuloma, LN lymph node. Source data are provided in Supplementary Data

On the basis of these results, we hypothesized that pulmonary infection with Mtb leads to the migration to and/or expansion of TRAV1-2^+^ CD8^+^ cells in the lung, potentially driven by Mtb-derived MR1 ligands. A hallmark of the human immune response to Mtb is the formation of lung granulomas. We therefore sought to determine the relevance of TRAV1-2^+^ T cell receptor (TCR) usage in lung granulomas from patients with TB. Single cell suspensions were prepared from diseased lung parenchyma from individuals (*n* = 5) undergoing clinically indicated surgical resection for complications of TB^18^. The most highly diseased lung granuloma (LG) tissues were designated “A” and the least diseased tissues designated “C.” CD4^-^ T cells from these samples were sorted by flow cytometry and subjected to high-throughput repertoire analysis using the bias-controlled immunoSEQ TCR sequencing platform^19^. In the 12 samples that yielded the minimal necessary sequencing data for analysis (>10^4^ productive reads, yielding a median of 3,919 unique productive TCRα reads (range 397–28,792) and a median of 167 TRAV1-2-utilizing unique productive TCRα reads (range 19–1081), the overall frequency of TRAV1-2^+^ TCR sequences in granulomas ranged from 3.1 to 5.9% across all donors and tissue samples (Fig. 2a and Supplementary Table 1). These frequencies are similar to those observed in peripheral blood and lymph nodes. We then developed an algorithm based on published MAIT CDR3α amino acid (aa) sequences^16,20^ to determine which of these TRAV1-2^+^ CDR3α sequences represented MAIT cell-consistent TCRαs. A CDR3α sequence similarity analysis was performed using “MAIT Match” (http://www.cbs.dtu.dk/services/MAIT_Match), a tool based on the method described by Shen et al.^21^, where a score of 1 reflects a perfect match and a score of 0 a perfect mismatch with published MAIT cell CDR3α sequences. To determine the validity of this tool, we compared the proportion of TRAV1-2^+^ sequences with the proportion of TRAV12-2^+^ sequences (an unrelated control) for TCRs with scores ranging from 0.85 to 1. MAIT Match scores of 0.95 to 1 were significantly increased among the in TRAV1-2^+^ but not TRAV12-2^+^ TCR sequences (*P* = 0.0035, *P* = 0.00046, *t* test; Fig. 2b). We therefore chose a MAIT Match score of 0.95 as a conservative threshold to define MAIT cell-consistent TCRs (Fig. 2b). In one individual with paired samples from the lung and mediastinal lymph node (LN), TRAV1-2 usage was comparable at both sites, but similarity analysis revealed MAIT cell-consistent TCR enrichment in the lung (*P* < 0.0001; 2-way ANOVA; Fig. 2c).

To address the possibility that Mtb drives the recruitment and/or expansion of TRAV1-2^+^ T cells with MAIT-consistent CDR3α‘s in granulomatous tissue, we analyzed the MAIT cell-consistent CDR3α sequences (MAIT Match score 0.95-1) found in diseased lung parenchyma (*n* = 5 individuals, 11 samples). It is established that certain MAIT cell TCRα chains can be shared among individuals (public sequences)^22^, while donor-unique (private) CDR3α sequences can be selected in response to distinct microbes^16^. As shown in Fig. 2d, both private and public CDR3α sequences were detected among the MAIT cell-consistent CDR3α sequences present in granulomatous lung tissue isolated from patients with TB. Notably, public MAIT cell-consistent CDR3α were frequently encoded by multiple synonymous nucleotide sequences within individuals suggesting the expansion of multiple clones with the same CDR3α amino acid sequences (Fig. 2e, right). In contrast, private MAIT cell-consistent CDR3α sequences were encoded by individual nucleotide sequences suggesting that these were the result of expansions of a single MAIT cell clone in each donor (Fig. 2e, left). Private CDR3α sequences were not restricted to infrequent clonotypes and in some tissue samples occurred as the dominant MAIT cell-consistent TCR.

### Bronchoalveolar TRAV1-2^+^ CD8^+^ T cells in active pulmonary TB

**Fig. 3:**
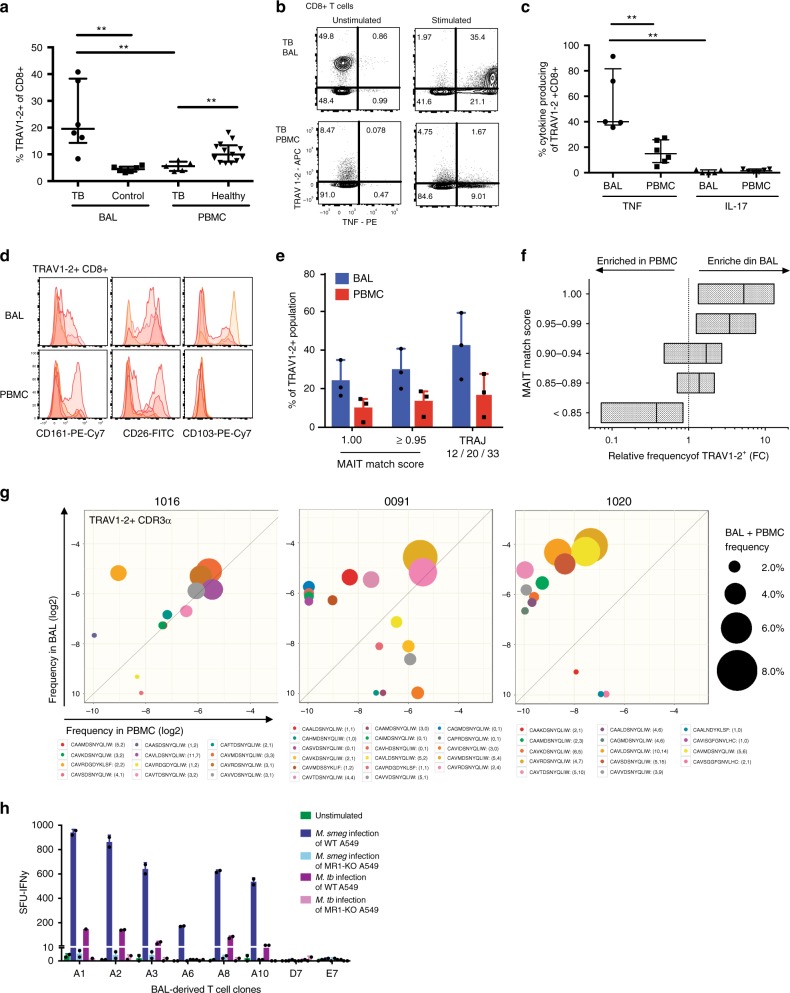
TNF-producing TRAV1-2^+^ CD8^+^ cells including oligoclonally expanded MAIT cells are enriched in bronchoalveolar lavage fluid from patients with TB. **a** Frequency of TRAV1-2^+^ cells among CD8^+^ T cells from the bronchoalveolar lavage (BAL) fluid from patients with TB (*n* = 6 biologically independent samples) and cancer controls (*n* = 6 biologically independent samples), and among CD8+ T cells in matched peripheral blood samples (PBMC) from patients with TB (*n* = 5 biologically independent samples) and unmatched peripheral blood samples from healthy controls (*n* = 13 biologically independent samples). Medians and interquartile ranges are displayed. **P < 0.01; Mann–Whitney *U* test. **b** Dot plots showing TNF production by TRAV1-2^+^ CD8^+^ T cells in matched BAL and peripheral blood samples (PBMC) from a patient with TB. Cells were stimulated with α−CD2/CD3/CD28 beads. **c** Frequency of TNF or IL-17 production by TRAV1-2^+^ CD8^+^ T cells in matched BAL and peripheral blood samples (PBMC; *n* = 5 biologically independent samples). Medians and interquartile ranges are displayed. **P < 0.01; Mann–Whitney *U* test. **d** Expression of CD161, CD26 and CD103 on TRAV1-2^+^ CD8^+^ T cells in matched BAL and peripheral blood samples (PBMC) from patients with TB (*n* = 4 biologically independent samples). Histograms are mode-normalized. **e** Frequency of MAIT cell-consistent CDR3α sequences within TRAV1-2^+^ CD4^-^ T cells in BAL fluid and peripheral blood samples (PBMC) from patients with TB (*n* = 3 biologically independent samples). Height represents the mean, error bars represent the range. **f** Relative frequency of CDR3α sequences by MAIT Match Score category in BAL fluid *vs*. matched peripheral blood (PBMC; *n* = 3 biologically independent samples). **g** Depiction of the top 10 most frequent MAIT cell-consistent CDR3α sequences (MAIT Match score ≥0.95) among TRAV1-2^+^ sequences in each compartment. Legend format: CDR3α aa (# of synonymous nucleotide sequences in peripheral blood, # of synonymous nucleotide sequences in BAL fluid). **h** IFNγ spot-forming units (SFU) produced by BAL T cell clones stimulated with *M. smegmatis*-infected or Mtb-infected wildtype (WT) or MR1-KO A549 cells (n=8 biologically independent clones). Height represents the mean of two independent replicates per stimulation, error bars represent the standard deviation. Source data are available in Supplementary Data.

Diminished frequencies of circulating MAIT cells have consistently been observed in people with TB^4,5^. This apparent peripheral depletion may occur as a consequence of selective MAIT cell migration to the lung or may reflect increased host vulnerability to infection with Mtb. Having found that TRAV1-2^+^ CD8^+^ cells are enriched in healthy airways and respond to mycobacteria (Fig. 1e)^4^, we hypothesized that pulmonary infection with Mtb drives the accumulation and expansion of TRAV1-2^+^ CD8^+^ cells in the lung in response to Mtb-derived MR1 ligands. To address this possibility, we measured the frequency of TRAV1-2^+^ CD8^+^ T cells in bronchoalveolar (BAL) fluid samples obtained from individuals with untreated, active pulmonary TB and controls with no evidence of infectious or inflammatory pulmonary disease (Supplementary Table 2). In BAL fluid, TRAV1-2^+^ CD8^+^ T cells were significantly enriched in patients with TB at frequencies approximately 3-fold higher than controls (*P* = 0.0022, Mann–Whitney *U* test, Fig. 3a). Conversely, in matched peripheral blood samples, TRAV1-2^+^ CD8^+^ T cells were significantly diminished in patients with TB at frequencies approximately 2-fold lower compared to healthy controls (*P* = 0.0028, Mann–Whitney *U* test, Fig. 3a). To assess the functional capacity of TRAV1-2^+^ CD8^+^ T cells in the BAL fluid and matched peripheral blood samples, we utilized α-CD2/CD3/CD28 beads as a stimulant to trigger responses via the TCR. Cell yields were insufficient to explore ligand-specific activation, which may also be subject to bias arising from compartment-specific differences in MR1-expression by antigen-presenting cells^23^. MAIT cells have been reported to produce IFN-γ, TNF, granzymes, granulysin, IL-17 and IL-22^24–26^. Among these, we chose to measure TNF, a representative Th1 effector cytokine essential for immune control of Mtb^27^ and IL-17, an immunomodulatory cytokine reportedly produced in a TCR-independent manner by MAIT cells^28^. A significantly greater proportion of TRAV1-2^+^ CD8^+^ T cells in BAL fluid produced TNF (median 40%, range 36–91%) compared with TRAV1-2^+^ CD8^+^ T cells in matched peripheral blood samples (median 15%, range 4.7–27%) (*P* = 0.004, Mann–Whitney *U* test, Fig. 3b, c and Supplementary Fig. 1). In contrast fewer than 1% of TRAV1-2^+^ CD8^+^ T cells in the BAL fluid and only 2% in matched peripheral blood samples produced IL-17 (Supplementary Fig. 2). We therefore concluded that TCR triggering of these BAL-resident TRAV1-2^+^ CD8^+^ T cells does not evoke IL-17 production, though other mitogenic or cytokine-associated stimulations may do so. Next, we characterized the phenotype of BAL-resident TRAV1-2^+^ CD8^+^ T cells. MAIT cells can be defined in peripheral blood by TRAV1-2 usage in conjunction with high-level expression of the c-type lectin CD161, and the di-peptidase CD26^13,26^. In BAL fluid obtained from patients with TB, TRAV1-2^+^ CD8^+^ T cells expressed low levels of CD161 compared with peripheral blood TRAV1-2^+^ CD8^+^ T cells (Fig. 3d), which is consistent with the data from healthy lung tissue (Fig. 1c) and the prior demonstration that CD161 can be down-regulated as a result of MAIT cell activation^17,28,29^. In contrast, TRAV1-2^+^ CD8^+^ T cells in the BAL fluid more consistently expressed CD26, which is abundantly present on all functional MR1-restricted MAIT cells in peripheral blood^17,24^. CD103, the αE integrin associated with tissue-resident memory T cells^30^ was expressed variably but exclusively on BAL TRAV1-2^+^ CD8^+^ T cells.

Although TRAV1-2 usage is a defining feature of MAIT cells, the same gene segment can be expressed by T cells recognizing mycobacterial ligands presented in the context of HLA-Ia molecules and CD1b^31^. On the basis that TRAV1-2^+^ CD8^+^ T cells display a surface phenotype suggestive of tissue-resident MAIT cells in BAL fluid isolated from patients with active TB, we postulated that the corresponding CDR3α sequences would provide a molecular signature reflecting MAIT cell enrichment relative to TRAV1-2^+^ CD8^+^ T cells in matched peripheral blood samples. To test this hypothesis, we performed high-throughput TCR repertoire analysis of TRAV1-2^+^ CD4^−^ T cells sorted by flow cytometry from cryopreserved BAL fluid and matched peripheral blood specimens obtained from three donors with active TB (Supplementary Table 3). MAIT cell-consistent CDR3α sequences comprised a higher percentage of the TRAV1-2^+^ repertoire in BAL fluid compared with peripheral blood, irrespective of the parameter used to define MAIT cell-consistent CDR3α sequences, including assessment of similarity to published MAIT cell CDR3α sequences (MAIT Match score = 0.95 or 1) or according to usage of TRAJ12, TRAJ20 or TRAJ33 (Fig. 3e; *P* = 0.0036; 2-way ANOVA). Among the patients with TB, CDR3α sequences with the highest MAIT Match scores (≥0.95) were enriched in BAL fluid, while those with the lowest MAIT Match scores (<0.85), were more frequent in peripheral blood (Fig. 3f ).

To determine the extent to which individual MAIT cell-consistent CDR3α sequences (MAIT Match Score ≥0.95) were shared between these two anatomical compartments, we created a TCR Enrichment Analysis (TEA) webtool (https://github.com/eisascience/Wong-Gold-Lewinsohn) to enable visualization and weighted frequency analysis of the most common MAIT cell-consistent CDR3α sequences in matched samples (Fig. 3g and Supplementary Table 4). In all three patients, the most frequent MAIT cell-consistent CDR3α sequences were present in both compartments, with disproportionate expansion in the BAL fluid compared with the peripheral blood. In contrast, CDR3α sequences with low MAIT Match scores (<0.85) were generally expanded only in one anatomical compartment (Supplementary Fig. 3). The selective expansion of MAIT cell-consistent CDR3α sequences in the lung compartment relative to peripheral blood suggests antigen-driven clonal expansion in response to pulmonary infection with Mtb.

To determine if TRAV1-2^+^ CD8^+^ T cells present in BAL fluid contained MAIT cells, we examined the MR1-restricted function of T cell clones generated from a BAL fluid sample obtained from a patient with TB. Six of these TRAV1-2^+^ clones (D0033-A1, A2, A3, A6, A8 and A10) expressed MAIT cell-consistent CDR3α sequences (MAIT Match score = 0.98-1; Table 1). Stimulation of these clones with HLA-mismatched *M. smegmatis*-infected or Mtb-infected antigen-presenting cells induced robust IFN-γ, while two control clones from the same patient (D0033-D7 and E7) failed to produce IFNγ under identical conditions. In contrast, stimulation of the TRAV1-2^+^ clones with HLA-mismatched *M. smegmatis*-infected or Mtb-infected MR1-KO antigen-presenting cells^32^ resulted in negligible IFN-γ-production, thereby demonstrating MR1-dependent cytokine production consistent with MAIT cell function (Fig. 3h).

**Table 1:** TCRα/β sequences and MAIT Match scores for six MAIT cell clones and two control clones derived from bronchoalveolar fluid from a patient with tuberculosis

Clone	TCRα sequencing				TCRβ sequencing		
	TRAV	CDR3α	TRAJ	MAIT Match score	TRBV	CDR3β	TRBJ
*MAIT cell clones*							
D0033-A1	1-2	CAALDSNYQLIW	33	1.00	4-3	CASSQDMVSITDTQY	2-3
D0033-A2	1-2	CAVTDSNYQLIW	33	1.00	3-1	CASSQAETELNTGELF	2-2
D0033-A3	1-2	CVTMDSNYQLIW	33	0.98	6-1	CASSEAGGGYNEQF	2-1
D0033-A6	1-2	CAVVDSNYQLIW	33	1.00	4-2	CASSHSSGTGGNEQF	2-1
D0033-A8	1-2	CVTMDSNYQLIW	33	0.98	6-1	CASSEAGGGYNEQF	2-1
D0033-A10	1-2	CAVTDSNYQLIW	33	1.00	3-1	CASSSGLEVTGELF	2-2
*Control T cell clones*							
D0033-D7	20	CAARFSDGQKLL	16	0.92	7-9	CASSEGTGVEWDGYT	1-2
D0033-E7	39	CAVPGGGADGLT	45	0.85	2	CASVASGVRDTQY	2-3

**Fig. 4:**
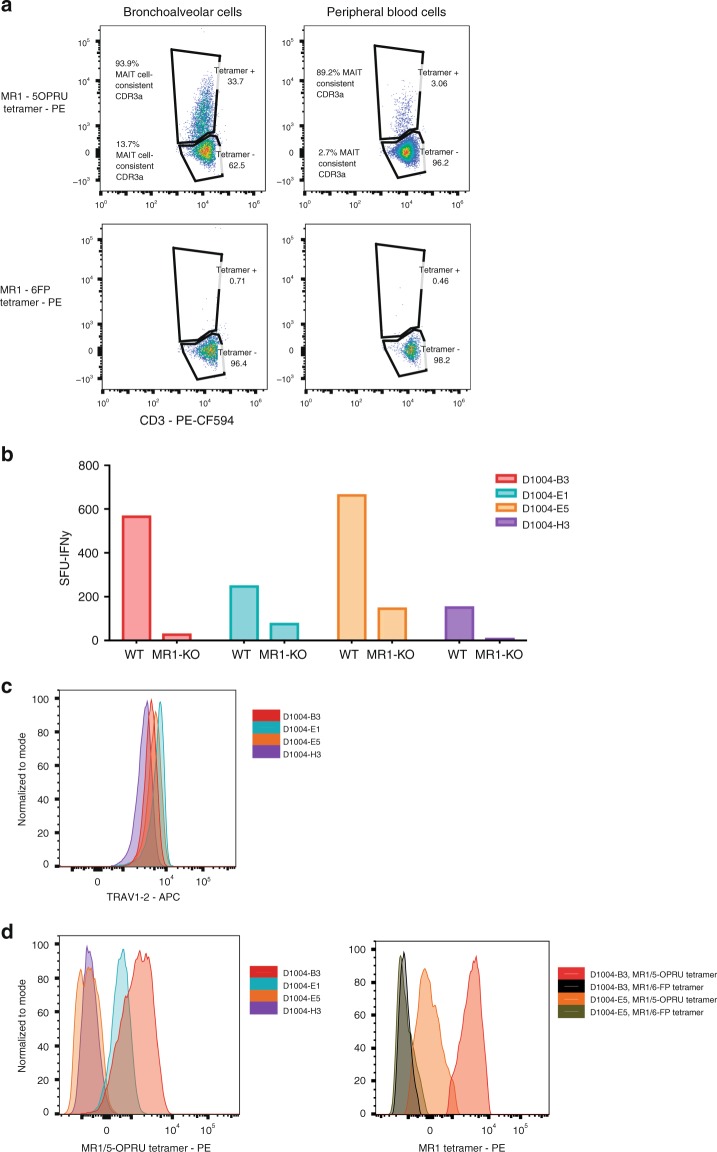
Heterogeneous MR1/5-OP-RU staining of bronchoalveolar TRAV1-2^+^ CD8^+^ T cells with MAIT cell-consistent CDR3α‘s and MR1-restricted function. **a** Frequency of MR1-tetramer^+^ cells (loaded with active (5-OP-RU) and control (6FP) ligand) in TRAV1-2^+^ T cells (gated on live, CD3^+^, CD8^+^ lymphocytes) from the BAL fluid and peripheral blood of a patient with TB. The proportion of cells utilizing MAIT cell-consistent CDR3α‘s (MAIT Match Score ≥.95) in MR1/5-OP-RU tetramer positive and negative populations are shown. **b** IFNγ spot-forming units (SFU) produced by four T cell clones generated from BAL fluid and stimulated with *M. smegmatis*-infected wildtype (WT) or MR1-KO A549 cells, Supplementary Data. **c** α-TRAV1-2 staining of four T cell clones generated from BAL fluid demonstrates consistent staining. Histograms are mode-normalized. **d** Binding of MR1/5-OP-RU tetramer on the same four T cell clones generated from BAL fluid demonstrates heterogenous MR1/5-OPRU tetramer staining (left). Binding of MR1/6-FP (control) and MR1/5-OPRU tetramer is shown for two clones (right). Histograms are mode-normalized

MR1 tetramer loaded with 5-OP-RU ligand has been shown to identify functional MAIT cells in the human peripheral circulation^13^. To evaluate whether TRAV1-2^+^ CD8^+^ T cells in the BAL of humans with TB could be stained by MR1/5-OP-RU tetramer, as well as the relationship between MAIT cell-consistent CDR3α usage and MR1/5-OP-RU tetramer staining, we took advantage of two donors with TB with available cryopreserved specimens. We stained cells from paired BAL and peripheral blood samples with TRAV1-2 antibody, MR1/5-OP-RU tetramer and MR1/6-FP tetramer (negative control). As shown in Fig. 4a, BAL cells from donor 1020 demonstrated MR1/5-OP-RU tetramer staining of 33.7% of the TRAV1-2^+^ cells, supporting the TCRα sequencing analysis that found that 40.7% of BAL TRAV1-2 CDR3α sequences were MAIT cell-consistent (MAIT Match score >.95). In the peripheral blood of this participant, only 3.06% of the TRAV1-2^+^ peripheral cells demonstrated MR1/5-OP-RU tetramer staining, in line with the TCRα sequencing analysis that had found that 5.09% of peripheral TRAV1-2 CDR3α‘s were MAIT cell-consistent.

In contrast to MR1/5-OP-RU tetramer staining in the peripheral blood where positive and negative populations were clearly discernable, the MR1/5-OP-RU tetramer staining of BAL cells was of heterogeneous intensity and did not allow unambiguous delineation of MR1/5-OP-RU tetramer negative and positive populations. As a result, we sorted TRAV1-2^+^ cells based on MR1/5-OP-RU tetramer staining, subjected both positive and negative subsets to TCR sequencing, and analyzed MAIT cell-consistent CDR3α usage in each population (Table 2). CDR3α chain sequencing of MR1/5-OP-RU tetramer positive cells from BAL and peripheral blood revealed that 93.9% and 89.2% of these respectively utilized MAIT cell-consistent TCRs. CDR3α chain sequencing of the MR1/5-OP-RU tetramer negative TRAV1-2^+^ populations demonstrated that a substantial proportion (13.7%) of the MR1/5-OP-RU tetramer negative cells in the BAL utilized MAIT cell-consistent CDR3α chains. In contrast, only 2.7% of MR1/5-OP-RU tetramer negative cells from the peripheral blood utilized MAIT cell-consistent CDR3α chains. These data suggest that MR1/5-OP-RU tetramer may perform less efficiently in BAL fluid than in peripheral blood. Notably, in the other donor (91), in whom 28.5% of the TRAV1-2^+^ cells had a MAIT cell-consistent CDR3α, only 5.09% of the TRAV1-2^+^ cells from the BAL stained MR1/5-OP-RU positive. In this donor, 24.7% of the MR1/5-OP-RU tetramer negative cells had MAIT cell-consistent CDR3α chains, suggesting that MR1/5-OP-RU tetramer staining of BAL cells may underestimate the presence of MAIT cells as determined by CDR3α usage.

**Table 2:** Comparison of MR1/5-OPRU tetramer staining and usage of MAIT cell-consistent CDR3α sequences within TRAV1-2^+^ CD8^+^ T cells in the bronchoalveolar (BAL) and peripheral blood (PBMC) compartments from two patients with active TB

Participant ID	Compartment	% MAIT cell-consistent CDR3α	% MR1/5-OPRU tetramer-positive	% MAIT cell-consistent CDR3α of MR1/5-OPRU tetramer-positive	% MAIT cell-consistent CDR3α of MR1/5-OPRU tetramer-negative
1020	BAL	40.7	33.7	93.9	13.7
1020	PBMC	5.4	3.1	89.2	2.7
0091	BAL	28.5	5.1	100.0	24.7
0091	PBMC	18.6	6.3	71.4	15.1

To better understand the relationship between MR1/5-OP-RU tetramer staining, CDR3α usage and MR1-dependent T cell activity, we sorted MR1/5-OP-RU positive cells from the BAL of an available individual with non-TB pneumonia and performed limiting dilution cloning using anti-CD3 and IL-2 stimulation. Following rapid expansion^33^, each clone was characterized functionally for MR1-restriction and antigenic specificity. As shown in Fig. 4c, four clones (D1004-B3, E1, E5, and H3) produced IFN-γ when stimulated with *M. smegmatis*-infected antigen-presenting cells (A549) and abrogated IFN-γ production when stimulated with identically infected MR1-KO antigen-presenting cells^32^. TCR sequencing demonstrated that each of these clones utilized a MAIT cell-consistent CDR3α (Fig. 4b). Surprisingly, despite clear evidence of MR-1 restricted function, usage of MAIT cell-consistent CDR3αʼs (Table 3) and TRAV1-2 staining of similar intensity (Fig. 4c), these clones demonstrated considerable heterogeneity in MR1/5-OP-RU tetramer staining, with two of the four clones staining weakly (Fig. 4d).

**Table 3:** TCRα/β sequences and MAIT Match scores for four MAIT cell clones derived from bronchoalveolar cells

Clone	TCRα sequencing				TCRβ sequencing		
	TRAV	CDR3α	TRAJ	MAIT Match score	TRBV	CDR3β	TRBJ
D1004-B3	1-2	CAVTDSNYQLIW	33	1.00	6-5	CASSYEGGGQPQHF	1-5
D1004-E1	1-2	CAALDSNYQLIW	33	1.00	6-4	CASSDGEGQPQHF	1-5
D1004-E5	1-2	CAAMDSNYQLIW	33	1.00	30-1	CAWSHSDRDLNEQYF	2-7
D1004-H3	1-2	CAAMDSNYQLIW	33	1.00	3	CASSQASGGEETQYF	2-5

## Discussion

Collectively, our data indicate that donor-unrestricted mycobacterial-reactive TRAV1-2^+^ CD8^+^ T cells are present in the human respiratory mucosa and that pulmonary infection with Mtb leads to an enrichment of airway resident, pro-inflammatory TRAV1-2^+^ CD8^+^ cells including oligoclonal expansions of MAIT cells.

In lung tissue explanted from healthy organ donors, we find that TRAV1-2^+^ CD8^+^ T cells localize to the respiratory tract mucosal surface. In contrast to their counterparts in the gut mucosa, TRAV1-2^+^ CD8^+^ T cells from the respiratory mucosa produce TNF in response to mycobacterial stimulation by donor-unrestricted antigen-presenting cells. This suggests that TRAV1-2^+^ CD8^+^ T cells in the airway mucosa may play a role in anti-Mtb immunity by initiating a local pro-inflammatory response upon exposure to aerosolized Mtb.

In the setting of active pulmonary tuberculosis, we observed striking expansions of TRAV1-2^+^ CD8^+^ T cells in the bronchoalveolar compartment. Compared to paired peripheral blood TRAV1-2^+^ CD8^+^ T cells, the bronchoalveolar TRAV1-2^+^ CD8^+^ T cells produced significantly more TNF. Some, but not all, of these expanded bronchoalveolar TRAV1-2^+^ CD8^+^ T cells could be identified as MAIT cells based on their utilization of MAIT cell-consistent CDR3α chains, demonstration of MR1-restricted function or selective binding of the MR1/5-OP-RU tetramer. It should be noted that among the TRAV1-2^+^ CD8^+^ T cells that could not be unequivocally confirmed as MAIT cells we identified subpopulations that displayed certain “MAIT-like” features, such as high-level expression of CD26 or oligoclonal expansions of TRAV1-2^+^ TCRα chains with features similar to MAIT cell CDR3α sequences (incorporation of the TRAJ12, TRAJ20 or TRAJ33 segments, or the presence of the Tyr95 which is known to be critical for MAIT cell TCR binding to MR1-restricted ligands^34^). Our attempts to clone these populations have been unsuccessful to date, such that further work will be required to determine if these TRAV1-2^+^ CD8^+^ T cells with “MAIT-like” features are restricted by MR1. It is also notable that MR1/5-OP-RU tetramers identified only a subset of TRAV1-2+ CD8+ T cells with MAIT cell-consistent CDR3a’s.

We postulate that variable MR1-tetramer staining observed on bronchoalveolar TRAV1-2^+^ CD8^+^ cells could reflect a state of activation among tissue-resident cells. Supporting this, we note that differential tetramer staining can be observed following expansion of MAIT cell clones with activating cytokines (Supplementary Figure 4). Alternatively, we postulate that TRAV1-2^+^ CD8^+^ T cells with MAIT cell-consistent CD3α‘s may have altered tetramer-binding avidity as a result of differential affinity of their TCRs for MR1-ligands. This possibility is suggested by the variable magnitude of response to *M. smegmatis* in the functional assay, and has recently been demonstrated for the photolumazine I ligand^35^. Further work will be required to better understand the relationship between TCR-dependent MR1-dependent activation, MR1/5-OP-RU tetramer staining, and ligand selectivity among bronchoalveolar TRAV1-2^+^ CD8^+^ T cells. At this point we conclude that MR1/5-OP-RU tetramer staining of bronchoalveolar MAIT cells is weaker and more variable than MR1/5-OP-RU tetramer staining of peripheral blood MAIT cells and hence may underestimate MAIT cell prevalence in the BAL.

In contrast to the bronchoalveolar fluid of active TB patients, analysis of TCRα chain usage in granulomas of patients undergoing lung-resection for clinically complicated tuberculosis did not demonstrate dramatic expansions of TRAV1-2^+^TCRαʼs. The contrast between the enrichment of TRAV1-2^+^ CD8^+^ T cells observed in bronchoalveolar lavage fluid and the relatively low frequencies of TRAV1-2^+^TCRα's found in the lung granuloma tissue may be due to differences between cells present in the airway mucosal environment and in lung parenchymal tissue. It is also possible that the kinetics of expansion of TRAV1-2^+^ CD8^+^ cells with MAIT cell-consistent CDR3α‘s varied during the long course of TB disease and anti-tuberculosis therapy that preceded surgical treatment in these medically-complex lung-resection patients.

It is therefore notable that even in the resected granuloma tissue, the subset of TCRα‘s with MAIT cell-consistent sequences was enriched among the TRAV1-2^+^ CDR3α‘s in lung granuloma tissue compared to paired mediastinal lymph node tissue. We postulate that this relative enrichment of MAIT cell-consistent TCRs among TRAV1-2^+^ sequences from the lung was driven by local antigen exposure, while acknowledging that tissue-specific non-antigen stimuli could also lead to the independent expansion of clones in the lung compartment. Further understanding of this will require additional organ-specific datasets to allow comparison of diseased and reference TCR repertoires. We found both public and private MAT cell-consistent CDR3α chains in the TB-infected human lung tissues we analyzed. Interestingly, public MAIT cell-consistent CDR3α chains were frequently encoded by multiple synonymous nucleotide sequences within an individual sample. This finding is consistent with a previous report implicating convergent recombination as a determinative process in the generation of public MAIT cell CDR3α sequences^22^, and suggests that tissue-resident public MAIT cell-consistent CDR3α expansions are the result of multiple individual MAIT cells clonally expanding in infected tissues. The significance of cells with private MAIT cell-consistent CDR3α chains in the context of Mtb-infected tissue remains uncertain. One possibility is that public and private MAIT cell-consistent CDR3α chains have similar ligand-binding properties, such that utilization and expansion of specific clonotypes in individual hosts is the result of differences in the naive TCR repertoire and is not driven by specific microbial exposures. Alternatively, the observed clonal expansion of private MAIT cell-consistent clonotypes within Mtb-infected tissue may reflect selective expansions in response to the local presence of microbe-derived ligands presented by MR1^35^. A third possibility is that because our sample set is small, sequences that appear to be private in this analysis could in fact turn out to be public when larger numbers of individual donors are sampled. In order to determine the significance of private and public MAIT cell-consistent TCRs in the context of mycobacterial infection, further study of selective ligand specificity in larger numbers of donors is needed. Nonetheless, the convergence of multiple nucleotide rearrangements on expanded public MAIT cell-consistent TCRα chains suggests that in some instances, multiple MAIT cells with genetically unique but functionally similar TCRα chains clonally expand in the TB-infected lung, potentially in response to microbe-derived antigenic-stimulation.

In line with recent studies that have found MAIT cell expansions in the lungs of mice experimentally infected with Mtb^36^ and *S. enterica* serovar Typhimurium *(S*. Typhimurium)^37^, we found expansions of TRAV1-2^+^ CD8^+^ T cells with MAIT or MAIT-like features in the BAL and lung parenchyma in patients with TB. Supported by the findings Chen et al. who found that accumulation of MAIT cells in the lungs of mice following challenge with *S*. Typhimurium is dependent on antigen derived from the microbial riboflavin synthesis pathway^37^, we postulate that these TRAV1-2^+^ CD8^+^ cell enrichments contain MAIT cells and are driven by Mtb*-*derived small molecular ligands. Howson et al. recently reported that MAIT cell clones with more avid ligand-binding expand during *S. enterica* serovar Paratyphi A infection and that these clones remain expanded after treatment of the infection^38^. This finding supports the idea that exposure to microbe-derived MR1 ligands alters the human MAIT cell TCR repertoire and suggests a role for MR1-ligand vaccine strategies. Overall our findings suggest a previously unrecognized and potentially important role for TRAV1-2^+^ CD8^+^ T cells with MAIT or MAIT-like features in the immune response to aerosolized Mtb infection, and would support exploration of these cells as targets of either vaccination or immunotherapeutic strategy.

## Methods

### Human subjects

*Samples from Portland, Oregon, USA*. Airway, lung, small intestine and associated lymph node tissues ineligible for transplantation were obtained from the Pacific Northwest Transplant Bank under a protocol approved by the Institutional Review Board at Oregon Health & Science University. Limited clinical information was available for these individuals, who were generally considered healthy prior to demise. For comparison with the organ samples, PBMCs were obtained by apheresis from healthy adult donors providing informed consent.

*Samples from Durban, South Africa*. Explanted granulomatous lung tissue and associated lymph nodes were obtained under a protocol approved by the University of KwaZulu Natal Human Biomedical Research Ethics Commitee (UKZN BREC) allowing adults undergoing clinically indicated lung resection for complicated tuberculosis at Inkosi Albert Luthuli Central Hospital (IALCH) to donate excess tissue for scientific research^18^. Tissue was isolated from different areas of resected lungs based on the experience of the operating surgeon and the preoperative radiological data. Clinical characteristics of the individuals and samples have been described^18^. All donors provided written informed consent prior to surgery. BAL fluid and paired peripheral blood samples were obtained under a protocol approved by the UKZN BREC and Partners Institutional Review Board allowing collection of excess fluid from adult patients undergoing clinically indicated diagnostic bronchoscopies at IALCH. Active tuberculosis was defined microbiologically (positive BAL Mtb culture or BAL Mtb PCR by GeneXpert) and/or histologically (Ziehl-Neelsen positive necrotizing granulomas on transbronchial biopsy obtained at the time of BAL). Uninfected controls were defined as individuals with no evidence of either infectious or inflammatory lung disease, as determined by a committee of study physicians on the basis of clinical history, chest x-rays, computerized tomography scans, and negative BAL microbiology (mycobacterial, bacterial and fungal cultures, and Mtb PCR). Most controls underwent bronchoscopy for suspected lung cancer, and a non-cancerous segment was lavaged in these cases. All donors provided written informed consent prior to bronchoscopy. Cryopreserved peripheral blood mononuclear cells (PBMCs) from healthy donors (defined as asymptomatic and HIV-negative with no evidence of Mtb by ELISPOT) were available from the iThimba Cohort which was approved by the UKZN BREC and Partners Institutional Review Board^39^. All participants provided written informed consent.

### Isolation and stimulation of lung and gut T cells

Lymphocytes were isolated from fresh lung tissue as described previously^4^. A two-step process was used to extract cells from the small intestine. For collection of lymphocytes from the intraepithelial (IEL) layer, the tissue was washed in HBSS, stripped of muscle, and incubated with agitation for 30 min in 0.15% dithiothreitol (Sigma-Aldrich). IEL lymphocytes were then harvested, and the remaining tissue was incubated for 30 min in PBS. Lamina propria (LP) lymphocytes were released by digestion with 0.1% collagenase (CLS-3, Worthington) and 0.3% DNAse (Roche) for 30 min at 37 °C. IEL and LP preparations were further enriched over a discontinuous Percoll gradient. Lymphocyte stimulations were performed as described previously^4,12,16^. Briefly, lymphocytes were incubated for 16 h with uninfected (control) or *M. smegmatis* strain mc^2^122-infected (multiplicity of infection = 3) A549 cells (ATCC CCL-185) at a ratio of 3:1 in the presence of α-CD28 and α-CD49d (Biolegend), together with an α-TNF mAb (Beckman Coulter) and the TNF-Processing Inhibitor 0 (TAPI-0, 10 μM) (Calbiochem). Cells were then stained as described above for surface expression of CD45, CD3, CD8, TRAV1-2, and CD161^4^. Dead cells were excluded using Aqua LIVE⁄DEAD (Invitrogen). Stained samples were acquired on a Fortessa flow cytometer (BD Biosciences) and data were analyzed with FlowJo software version 10.6 (Tree Star).

### Immunohistochemistry of airway tissues

Cryosections (10 µM) of frozen airway tissues were treated with acetone and air-dried prior to incubation with α-TRAV1-2 antibody (clone-3C10; Biolegend) followed by goat α-mouse IgG1-Alexa Fluor 488 (1:1000), and then α-CD8 antibody (1:50; LSBio) followed by goat α-mouse IgG1-Alexa Fluor 568 (1:1000). Sections were washed and stained with DAPI. Images were acquired using an Olympus FluoView FV1000 laser scanning confocal microscope system with a 40 × 1.3 Oil Plan Fluorite objective. Confocal images were analyzed using Imaris Analysis Software.

### Isolation and TCR sequencing of T cells from lung granulomas

Diseased lung tissue (approximately 3 cm^3^) was isolated from surgically resected explants. Each sample was washed in multiple changes of Hank’s Balanced Salt Solution (HBSS), diced into smaller pieces (approximately 1 mm^3^), strained, resuspended in pre-warmed R10 supplemented with 0.5 mg/ml collagenase D (Roche) and 40 U/ml DNaseI (Roche), and transferred to GentleMACS C-tubes (Miltenyi Biotec) for mechanical digestion per the manufacturer’s instructions. The resulting suspension was incubated for 60 min at 37 °C, subjected to an additional mechanical digestion step, strained through a 70 μm filter, washed twice in HBSS, and stained prior to sorting CD^4−^ T-cells using a FACSARIA flow cytometer (BD Biosciences). Cells were gated as live (nearIR^−^, Invitrogen), single lymphocytes (determined on the basis of light scatter), then sorted as CD45^+^, CD3^+^, CD4^−^ events directly into RLT buffer. Genomic DNA was extracted using a DNeasy Minikit (Qiagen) and high-throughput TCRα sequencing was performed using the ImmunoSEQ assay (Adaptive Biotechnologies Corp.)^40^. Data were analyzed using the ImmunoSEQ Analyser.

### CDR3α sequence similarity

Similarity between CDR3α sequences was calculated as described previously^21^. This method allows similarities to be assigned between sequences of different length in an alignment-free manner. An implementation of the similarity matching between CDR3 sequences is publicly available at http://www.cbs.dtu.dk/services/MAIT_Match. The server takes as input a list of CDR3α sequences, and returns for each a score based on the maximal sequence similarity with a reference database of MAIT cell CDR3α sequences. A perfect match has a similarity score of 1, and a perfect mismatch a similarity score of 0.

### Collection, staining and stimulation of BAL lymphocytes

Bronchoscopies were performed by pulmonologists at IALCH. Patients received sedation and bronchodilators according to the local standard of care; Normal saline (200 mL) was lavaged into the lobe with the highest burden of pathology or, in patients with diffuse disease, the right middle lobe. Available BAL fluid was combined in a 1:1 ratio with R10 (RPMI 1640 supplemented with 10% fetal bovine serum, L-glutamine, penicillin and streptomycin) and stored on ice. All samples were processed within 3 h of collection, BAL fluid was filtered through a 40 µm strainer (BD Pharmingen) and centrifuged. Resuspended BAL cells were aliquoted for staining with Aqua LIVE⁄DEAD (Invitrogen) and some or all of the following antibodies: α-CD3-PE-CF594 (BD Horizon, clone UCHT1), α-CD8-APC-H7 (BD Pharmingen, clone SK1), α-CD14-PerCP-Cy5.5 (BioLegend, clone HCD14), α-CD235a-PerCP-Cy5.5 (BioLegend, clone HIR2), α-TRAV1-2-APC (clone OF-5A12^12^), α-CD161-PE-Cy7 (BioLegend, clone HP-3G10), α-CD26-PE (BioLegend, clone BA5b). All stains were performed at 4 °C. Cells were then fixed with 4% paraformaldehyde. Functional studies were performed if sufficient numbers of BAL lymphocytes were available. After depletion of macrophages via plastic adherence for 1 h, 1 × 10^6^ lymphocytes were stimulated for 18 h at 37 °C with α-CD2/CD3/CD28-loaded Anti-Biotin MACSiBead Particles (Miltenyi Biotec) at a ratio of 2:1 in RPMI 1640 supplemented with 10% fetal bovine serum, L-glutamine, HEPES, penicillin, and streptomycin. Brefeldin A was added after the first hour to inhibit protein transport from the endoplasmic reticulum. Stimulated and unstimulated cells were then stained with Aqua LIVE⁄DEAD (Invitrogen) and the following antibodies: α-CD235a-PerCP-Cy5.5, α-CD14-PerCP-Cy5.5, α-CD8-APC-H7, α-TRAV1-2-APC, α-CD161-PE-Cy7. After a wash step, cells were fixed with PERM/FIX Medium A (Invitrogen), permeabilized with PERM/FIX Medium B (Invitrogen), and stained with the following antibodies: α-CD3-PE-CF594, α-TNFa-PE (Beckman Coulter, clone IPM2), and α-IL-17-BV421 (BioLegend, clone BL168). Stained samples were acquired using a Fortessa flow cytometer (BD Bioscience). Rainbow Fluorescent Particles (BD Bioscience) and applications settings in FACSDiva7 were used to correct for day-to-day variations in instrument performance. Cells were gated as live (aqua viability dye negative) lymphocytes (determined on the basis of light scatter), and CD14^++^ cells were excluded prior to selecting CD3^+^ cells for analysis. Paired peripheral blood samples were collected where possible and freshly isolated PBMC were processed in parallel with matched BAL cells. Data were analyzed with FlowJo10.6 (Treestar). Background cytokine production was subtracted to calculate percentage of cells producing cytokine in response to stimulation. When available, paired cryopreserved BAL and PBMC cells were thawed and stained with some or all of the above antibodies and MR1/5-OP-RU or MR1/6-FP tetramers (courtesy of the McCluskey Laboratory). Cell suspensions were acquired and sorted on a FACSARIA flow cytometer (BD Biosciences) into TRIzol (Invitrogen). Genomic DNA was extracted utilizing the phenol-chloroform method according to manufacturer protocol, using linear acrylamide (Invitrogen) as a carrier. High-throughput TCRα sequencing was performed using the ImmunoSEQ assay (Adaptive Biotechnologies Corp)^40^.

### Visualization of MAIT cell CDR3α sequences

Data were coded in R using the packages RColorBrewer, Shiny, data.table, ggplot2, and dplyr. Synonymous nucleotide sequences within a tissue were counted, and the associated frequencies are summed. These frequencies were visualized using the TCR Enrichment Analysis (TEA) webtool the code for which is archived at https://github.com/eisascience/Wong-Gold-Lewinsohn/tree/v1.0.0

### Generation and characterization of T cell clones

Cells from BAL samples were stained with Aqua LIVE/DEAD (Invitrogen), MR1/5-OP-RU tetramer (0.3 nM, McCluskey Laboratory), α-CD4-FITC (clone OKT4; BioLegend), and α-CD8-APC-Cy7 (clone SK8; BioLegend). Live tetramer-binding cells were sorted by the basis of co-receptor expression using an Influx flow cytometer (BD Biosciences), rested overnight in RPMI 1640 supplemented with 10% heat-inactivated pooled human serum and 0.5 ng/ml rhIL-2, and then distributed in limiting dilution format with irradiated PBMCs (150 × 10^5^/well) and irradiated B-lymphoblastoid cells (3 × 10^4^/well) in a 96-well round bottom plate. The cultures were stimulated with rhIL-2 (5 ng/ml), rhIL-12 (0.5 ng/ml), rhIL-7 (0.5 ng/ml), rhIL-15 (0.5 ng/ml) and α-CD3 (0.03 µg/ml). Clones were harvested after incubation for 20 days at 37 °C and assessed for clonality by flow cytometry, TCR sequencing, and MR1-restricted function by ELISPOT.

Nitrocellulose-backed multiscreen 96-well plates (Millipore) were coated overnight at 4 °C with a 10 µg/ml solution of α-IFNγ antibody (clone 1-D1K; Mabtech) in 0.1 M Na_2_CO_3_, 0.1 M NaHCO_3_, pH 9.6. The plate was washed three times with sterile PBS and blocked for 1 h at room temperature with RPMI 1640 containing 10% heat-inactivated pooled human serum. Uninfected, *M. smegmatis* mc^2^122-infected (multiplicity of infection = 3), or *M. tuberculosis* H37Rv-infected (multiplicity of infection = 30) wildtype or MR1-null^32^ A549 cells (1 × 10^4^/well) and clonal T cells (1 × 10^4^/well) were added and incubated overnight at 37 °C. The plates were then washed six times in PBS containing 0.05% Tween-20, incubated for 2 h at room temperature with a 1 µg/ml solution of α-IFNγ-biotin antibody (clone 7-B6-1; Mabtech) in PBS containing 0.5% bovine serum albumin and 0.05% Tween-20, washed again six times in PBS containing 0.05% Tween-20 followed by PBS alone, and developed using an AEC Vectastain Kit (Vector Laboratories). Spots were counted using an automated ELISPOT Reader System (Autoimmun Diagnostika GmbH).

### TCR sequence analysis of CD8+ T cell clones isolated from BAL fluid

For some clones, total RNA was extracted using an RNeasy Mini Kit (Qiagen). Unbiased amplification of all expressed *TRA* and *TRB* gene products was then conducted using a template-switch anchored RT-PCR with chain-specific constant region primers^41^. Amplicons were sub-cloned, sampled, sequenced and analyzed as described previously^42^. Gene usage was assigned according to the IMGT nomenclature. For other clones, genomic DNA was extracted using a DNeasy Mini Kit (Qiagen) and high-throughput TCRα and TCRβ sequencing was performed using the ImmunoSEQ assay (Adaptive Biotechnologies Corp)^40^. Data were analyzed using the ImmunoSEQ Analyser.

### Statistics and reproducibility

Statistical analyses were performed using Prism 6 (GraphPad Software Inc). The non-parametric Mann–Whitney *U* test was used to assess differences between groups unless indicated otherwise. All statistical tests were two-sided unless indicated otherwise. *P* values < 0.05 were considered significant for direct comparisons. In cases of multiple comparisons the Bonferonni correction was applied. Experiments were repeated with as many biologically indpendent samples as were available; when possible a minimum of two experimental replicates were performed.

### Reporting summary

Further information on research design is available in the Nature Research Reporting Summary linked to this article.

## Supplementary information

Supplementary Information

Reporting Summary

Supplementary Data

## Data Availability

The datasets generated during and/or analyzed during the current study are archived at https://github.com/eisascience/Wong-Gold-Lewinsohn or available from the corresponding author on reasonable request.

## Code availability

Custom code for the MAIT Match tool is available at http://www.cbs.dtu.dk/services/MAIT_Match/. Custom code for the visualization of frequencies of specific TCRα‘s in the TB granuloma tissues and the TCR Enrichment Analysis (TEA) webtool are archived at https://github.com/eisascience/Wong-Gold-Lewinsohn
