# Supplementary material for: TRAV1-2+ CD8+ T-cells including oligoconal expansions of MAIT cells are enriched in the airways in human tuberculosis
Source: Commun Biol. 2019 Jun 5;2:203. doi: 10.1038/s42003-019-0442-2 (PMC6549148; doi:10.1038/s42003-019-0442-2)
Supplement: Supplementary file 1 — Supplementary Information [file 42003_2019_442_MOESM1_ESM.pdf]

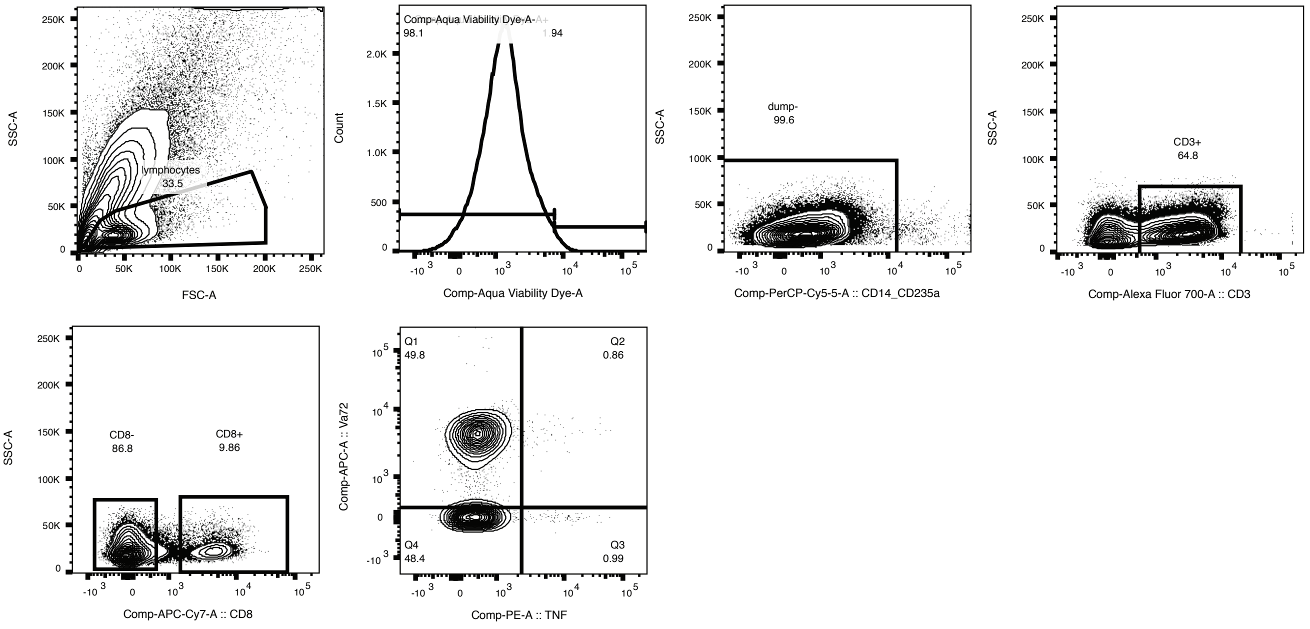

Supplementary Figure 1. Representative gating strategy for TRAV1-2+ CD8+ T cells from bronchoalveolar lavage fluid.

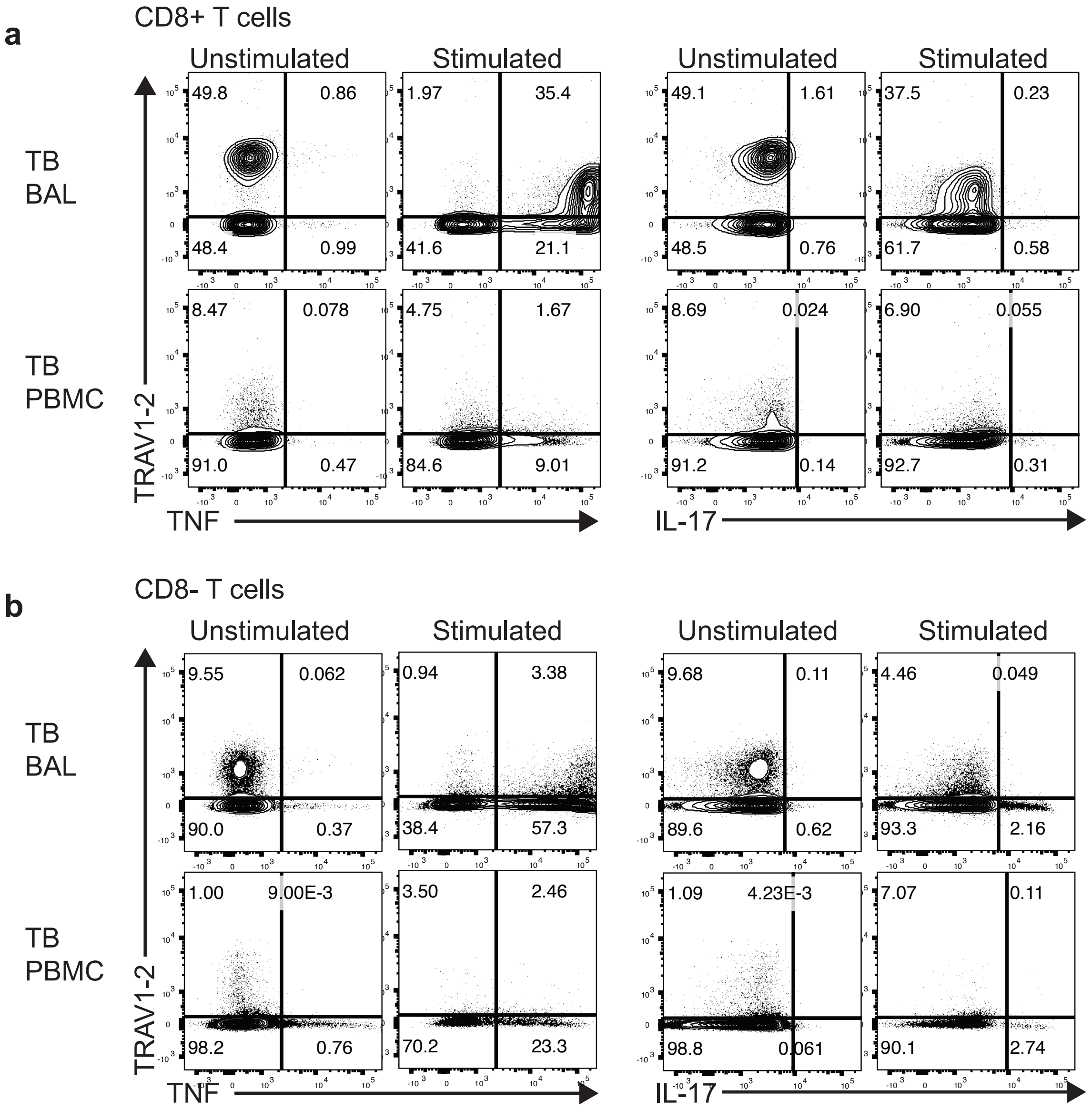

Supplementary Figure 2. Extended intracellular cytokine staining

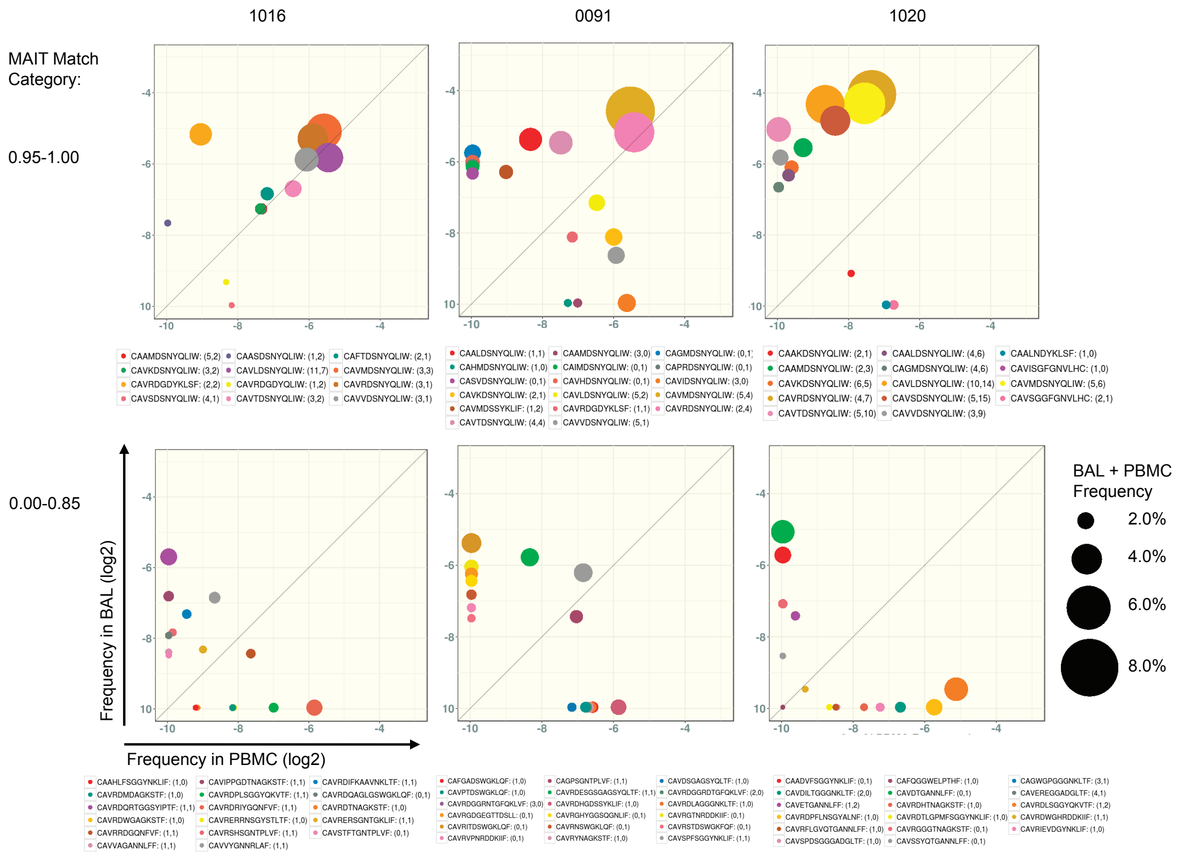

Supplementary Figure 3. Comparison of CDR3α sequences with MAIT Match scores > 0.95 vs. MAIT Match scores < 0.85.

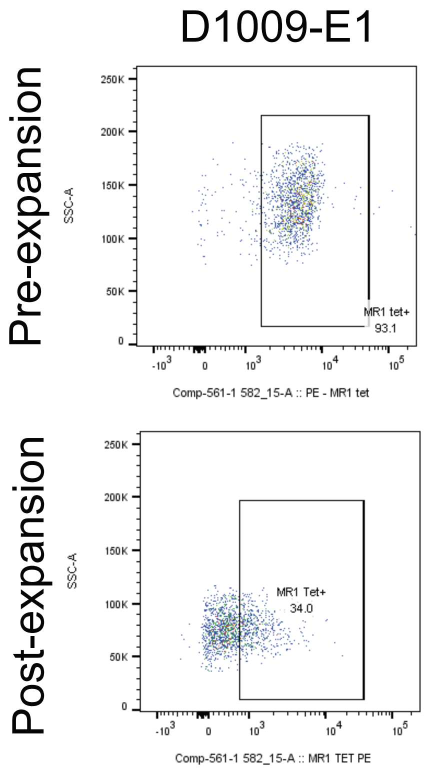

Supplementary Figure 4. MR1/5-OP-RU tetramer staining on MAIT cell clone D1009-E1, prior to and after expansion with T cell activating cytokines.

Supplementary Table 1. Yield of high-throughput repertoire analysis using the immunoSEQ TCR sequencing platform on lung granuloma samples from humans with TB infection.

Sample	Productive Total TCRA sequences	Productive Unique TCRA sequences	Productive Unique TCRA sequences featuring TRAV1-2
21LG	34542	928	31
23LG-A	39976	1241	80
23LG-B	258044	3932	188
23LG-C	95046	1908	110
23LN	555108	28782	1081
24LG-A	156138	3991	236
24LG-B	144975	4146	237
24LG-C	65982	1599	70
26LG-A	715503	16197	589
26LG-B	406067	10298	379
27LG-A	10006	397	19
27LG-B	202764	3905	145
minimum	10006	397	19
maximum	715503	28782	1081
median	150557	3919	167

Supplementary Table 2. Characteristics of tuberculosis patients and BAL controls. Fresh BAL and (for the tuberculosis donors) paired PBMC were used to generate the data presented in Figure 3a-c.

Study ID Number	Sex	Age	Race	HIV status	Diagnosis	Evidence for Tuberculosis Diagnosis
012-09-0011	M	59	Indian	Negative	Tuberculosis	AFB+ necrotizing granuloma on biopsy
012-09-0033	F	71	African	Negative	Tuberculosis	GeneXpert Mtb PCR and Mtb culture positive
012-09-0037	M	39	African	Negative	Tuberculosis	GeneXpert Mtb PCR and Mtb culture positive
012-09-0065	F	25	Indian	Negative	Tuberculosis	GeneXpert Mtb PCR and Mtb culture positive
012-09-0087	F	67	Indian	Negative	Tuberculosis	GeneXpert Mtb PCR and Mtb culture positive
012-09-0091	F	28	Indian	Negative	Tuberculosis	GeneXpert Mtb PCR and Mtb culture positive
012-09-0035	F	73	White	Negative	Lung Cancer	
012-09-0045	F	51	Indian	Negative	Lung Cancer	
012-09-0070	M	76	African	Negative	Lung Cancer	
012-09-0080	M	47	African	Negative	Lung Cancer	
012-09-0088	M	66	African	Negative	Lung Cancer	
012-09-0095	M	69	Indian	Negative	Lung Cancer	

Supplementary Table 3. Characteristics of tuberculosis patients whose cryopreserved paired BAL and PBMC samples were sorted to generate the data presented in Figure 3e-g.

Study ID Number	Sex	Age	Race	HIV status	Diagnosis	Evidence for Tuberculosis Diagnosis
012-09-0091	F	28	Indian	negative	Tuberculosis	GeneXpert Mtb PCR and Mtb culture positive
012-09-1020	M	19	African	positive, on ART	Tuberculosis	GeneXpert Mtb PCR and Mtb culture positive
012-09-1016	F	28	African	positive, on ART	Tuberculosis	GeneXpert Mtb PCR positive, Mtb culture negative

!

Supplementary Table 4. Top 15 MAIT CDR3α sequences in the bronchoalveolar lavage of humans with pulmonary tuberculosis

Bronchoalveolar Lavage - 0091

Frequency	CDR3a	TRAJ	MAIT Match Score
4.20%	CAVMDSNYQLIW	33	1.00
2.78%	CAVRDSNYQLIW	33	1.00
2.42%	CAALDSNYQLIW	33	1.00
2.27%	CAVTDSNYQLIW	33	1.00
1.86%	CAGMDSNYQLIW	33	1.00
1.55%	CAVHDSNYQLIW	33	0.96
1.44%	CAPRDSNYQLIW	33	0.95
1.41%	CAIMDSNYQLIW	33	1.00
1.28%	CAVMDSSYKLIF	12	1.00
1.25%	CASVDSNYQLIW	33	0.99
1.21%	CALMDSNYQLIW	33	0.98
1.01%	CAVTDSSYKLIF	12	0.98
1.00%	CASMDSNYQLIW	33	1.00
0.83%	CAVADSNYQLIW	33	0.98
0.81%	CAVNDGDYKLSF	20	0.95

Bronchoavleolar Lavage - 1016

Frequency	CDR3a	TRAJ	MAIT Match Score
2.95%	CAVMDSNYQLIW	33	1.00
2.78%	CAVRDGDYKLSF	20	1.00
2.55%	CAVRDSNYQLIW	33	1.00
1.77%	CAVLDSNYQLIW	33	1.00
1.70%	CAVVDSNYQLIW	33	1.00
0.97%	CAVTDSNYQLIW	33	1.00
0.87%	CAFTDSNYQLIW	33	0.96
0.65%	CAAMDSNYQLIW	33	1.00
0.65%	CAVKDSNYQLIW	33	1.00
0.50%	CAASDSNYQLIW	33	1.00
0.49%	CAVRDSDYKLSF	20	1.00
0.44%	CVAMDSNYQLIW	33	0.98
0.39%	CVTMDSNYQLIW	33	0.98
0.34%	CAYMDSNYQLIW	33	0.97
0.22%	CAVRDRDYQLIW	33	1.00

Bronchoavleolar Lavage - 1020

Frequency	CDR3a	TRAJ	MAIT Match Score
6.08%	CAVRDSNYQLIW	33	1.00
5.10%	CAVMDSNYQLIW	33	1.00
4.99%	CAVLDSNYQLIW	33	1.00
3.63%	CAVSDSNYQLIW	33	1.00
3.07%	CAVTDSNYQLIW	33	1.00
2.15%	CAAMDSNYQLIW	33	1.00
1.77%	CAVVDSNYQLIW	33	1.00
1.46%	CAVKDSNYQLIW	33	1.00
1.25%	CAALDSNYQLIW	33	1.00
0.99%	CAGMDSNYQLIW	33	1.00
0.85%	CAPMDSNYQLIW	33	1.00
0.64%	CASMDSNYQLIW	33	1.00
0.64%	CAVMDSSYKLIF	12	1.00
0.48%	CAVIDSNYQLIW	33	1.00
0.43%	CAGTDSNYQLIW	33	0.96
